# Practice of Dentistry in Foreign Countries

**Published:** 1898-03

**Authors:** 


### Editorial.

### PRACTICE OF DENTISTRY IN FOREIGN COUNTRIES.

The question is so frequently asked, "Upon what conditions can I enter practice in foreign countries?" that a brief statement of what a proposed resident in other lands must undergo may have its value to those who look with longing aspirations in that direction.

The extraordinary demand for the degree of Doctor of Dental Surgery has led to a great increase in the number of dentists in all our large centres of population, and the young aspirant for professional honors, feeling that all places are crowded in this country, seeks other fields to conquer. It is the old, old story of the gold-seeker. It was the Eldorado of the Spanish invaders, it is the Klondike of to-day, and professionally it is the foreign field where it is supposed the American dentist holds a pre-eminent position, and, being thus favorably situated, can command a practice and revenue largely in excess of his brethren less favorably circumstanced at home.

The idea that American dentistry is held at a premium in all foreign centres of civilization is so thoroughly engrafted on the dental mind through the labors of the eminent men who early braved all the difficulties and annoyances of expatriation, that it is almost a waste of time to endeavor to show that, whatever the financial returns may have been in the past, or even may exist in the present, they no longer, as a rule, are within the grasp of the graduate of to-day.

The tendency of all governments is towards protecting their own people. The competition in life has become so sharply concentrated that nationalities have felt forced to intrench themselves behind tariffs and restrictive laws for their own protection. We may inveigh against this as the product of the selfishness of the age; but while it is all this, it has its origin in the ever-existing fear of present and future financial injury.

The very fact that American dentistry secured recognition abroad raised an opposition in the minds of the members of the same profession in foreign lands. This was a natural result, and these men turned to law for protection in exactly the same way and for the same reasons that the residents of the several States in this country say to others of their countrymen, "This plot of ground is ours, and no man shall enter and reap the harvest except by our consent."

It has so happened that the writer of this has been consulted a score of times by young men anxious to go abroad, and invariably the answer has been given that it was unwise to attempt it.

The reasons for this advice are plentiful, but to the inexperienced rarely have weight. The first and most tangible difficulty, and can, in a measure, be appreciated, is inability to converse. To acquire a reasonable fluency in the use of a language is a matter of long practice and constant study; and until this is accomplished, at least in degree sufficient for daily use, success in securing and holding practice is not brilliant.

Presuming, however, that this really minor difficulty is overcome, and the aspirant for dental honors and emoluments has passed safely through the meshes of the legal nets thrown around him and has successfully sailed his bark into smoother seas, what has he to look forward to as the result of his labors? He may secure a large practice; he may even roll up a large bank account. He may marry and rear a family, and what then? He finds himself, as age creeps on, a man practically without a home. He may have all the beautiful surroundings of one, but, while an American at heart, he is no longer part of that free life he enjoyed in his youth. He has outlived everything once dear to him there, and has not become a part of the real life of the people among whom he is a worker. He is truly an exile from all that makes life worth the living. He sees his children growing up around him who know nothing of his early life or of its people. He would gladly make them Americans, but their young minds grow naturally into their surroundings. They speak his mother-tongue imperfectly, but are proficient in that of the country he inhabits. He cannot change the course of development of minds trained, as theirs must be, in the habits, customs, and the educational processes of the country; and so, in time, these children of his grow to be entirely uninterested in American life and its larger possibilities. The still darker prospect haunts him that the second generation will become citizens of the country he has learned to dwell in, but not to love.

While this is true of most countries, it is even worse in some, where it is absolutely impossible to rear children and educate them properly. These must be sent away thousands of miles to receive necessary mental training. The illustration of a father of a family toiling away in China, with that family in England, with no prospect of a reunion for years, is not a pleasant picture to dwell upon, and yet this is exactly the situation of one the writer recently met, and who sorrowfully stated the fact. Money failed to compensate in this case.

The idea that the financial returns are larger in some foreign city than at home is an error. The few favored men who have accumulated fortunes are the exceptions abroad, as they are the exceptions at home; but, were it all true, will money compensate for the deprivations to which allusion has been made?

This word of warning would seem almost useless, in view of the fact that the laws of nearly all countries are now in direct opposition to the practice of dentistry in foreign lands; yet, notwithstanding this difficulty, there is a class of young men, full of energy and adventurous spirit, who are willing to accept positions as assistants to American dentists abroad, and that without due consideration of the present and future difficulties they are sure to meet. For these the caution is extended, but that it will be regarded is not expected; for advice does not weigh in the balance with a salary in gold.

The anxiety to learn something of the laws regulating dentistry in foreign countries induced the Department of State at Washington to instruct the various consuls to report. These have been published, and through the kindness of the Secretary of State the writer is enabled to give abstracts of these reports upon another page. They will be found of interest to all, whether contemplating a permanent residence abroad or as instructors at home; for the latter are continually pressed for information in these directions at the graduating period. It will be observed that practically all doors are closed to the American dentist; and those contemplating entering its ranks must be made aware of the fact that the struggle for existence must be made in their own country; and this can best be carried out there amidst congenial surroundings, and in the end will insure a life of comfort, if not of luxury.

